# Construction and validation of a multimodal predictive model incorporating catecholamines and uric acid for early detection of hypertensive organ damage

**DOI:** 10.3389/fendo.2025.1687023

**Published:** 2025-10-15

**Authors:** Menglin Wang, Haiying Zhao, Dongyu Li

**Affiliations:** ^1^ Department of Hypertension, Henan Provincial People’s Hospital, Zhengzhou University People’s Hospital, Zhengzhou, China; ^2^ Department of Cardiovascular, Zhengzhou Hospital of Traditional Chinese Medicine, Zhengzhou, China

**Keywords:** hypertension, organ damage, catecholamine, serum uric acid, predictive model

## Abstract

**Objective:**

To investigate the feasibility and clinical value of constructing a predictive model for early detection of organ damage in hypertensive patients based on catecholamine-related indicators (norepinephrine, normetanephrine, and metanephrine), serum uric acid, and other clinical parameters.

**Methods:**

A total of 421 hypertensive patients were enrolled and divided into a training set (*n* = 295) and a validation set (*n* = 126) in a 7:3 ratio. Baseline data were collected, including catecholamine-related indicators (norepinephrine, epinephrine, normetanephrine, and metanephrine), serum uric acid, blood pressure parameters, target organ structural markers (left ventricular posterior wall thickness, carotid intima-media thickness, etc.), and clinical characteristics. Organ damage (defined as left ventricular hypertrophy, carotid intima-media thickness ≥1.0 mm, or elevated serum creatinine) was set as the outcome event. Univariate and multivariate logistic regression analyses were performed to identify independent predictors, followed by the construction of a nomogram model for performance evaluation and validation.

**Results:**

The incidence of organ damage was 44.07% (130/295) in the training set and 42.06% (53/126) in the validation set. Multivariate regression revealed that norepinephrine, normetanephrine, metanephrine, serum uric acid, serum creatinine, duration of hypertension, and cystatin C were independent predictors of organ damage (all *P* < 0.05). The nomogram model demonstrated C-indices of 0.834 and 0.823 in the training and validation sets, respectively, with AUCs of 0.834 (95% *CI*: 0.779–0.888) and 0.823 (95% *CI*: 0.732–0.914). Sensitivity and specificity were 0.717 and 0.819 in the training set and 0.711 and 0.776 in the validation set. Calibration curves indicated good agreement between predicted and observed values, with Hosmer-Lemeshow test *P*-values of 0.617 and 0.472, respectively.

**Conclusion:**

The predictive model constructed based on relevant indicators such as catecholamines and serum uric acid in this study can effectively predict the risk of organ damage in hypertensive patients, intervene early, and provide a quantitative basis for clinical decision-making.

## Introduction

Hypertension is one of the most prevalent chronic diseases worldwide, with an adult prevalence rate of 23.2% in China. The resultant target organ damage (TOD) (e.g., cardiac hypertrophy, renal impairment, carotid artery lesions) serves as a major driver of cardiovascular events and mortality (1, 2). Studies indicate that approximately 30%–50% of hypertensive patients develop at least one organ injury within five years of diagnosis, whereas early detection during the asymptomatic phase may reduce cardiovascular risk by 20%–30% (3, 4). Conventional organ damage detection relies on ultrasound and biochemical markers, yet these methods suffer from insufficient sensitivity or delayed detection (e.g., serum creatinine only shows significant elevation after >50% renal function loss).

Catecholamines, as key biomarkers of sympathetic nervous activity, contribute to target organ damage through mechanisms such as sustained vasoconstriction and enhanced oxidative stress due to metabolic dysregulation (e.g., elevated norepinephrine). Concurrently, serum uric acid (SUA) participates in renal and vascular injury via crystal deposition and inflammatory responses. Both factors have been widely validated in association with hypertensive organ damage (5–7). However, single biomarkers exhibit limited predictive efficacy (e.g., SUA alone yields an AUC of only 0.65–0.70 for renal impairment prediction) and lack integrated quantitative tools incorporating multidimensional indicators (8–10).

With the advancement of multimodal predictive frameworks, the construction of combined models integrating biomarkers and clinical parameters has emerged as a promising approach. This study aims to analyze the interactions among catecholamine derivatives, SUA, and clinical features to establish a visualized nomogram model, thereby improving early detection efficiency for hypertensive organ damage and supporting personalized risk management decision-making.

## Materials and methods

### Study population

A total of 421 hypertensive patients admitted between January 2023 and March 2025 were enrolled. Inclusion criteria: (1) Diagnosis per the Chinese Guidelines for Hypertension Management (2024 Revision) (11) (systolic blood pressure [SBP] ≥140 mmHg and/or diastolic blood pressure [DBP] ≥90 mmHg, or current antihypertensive medication use). (2) Age 18–80 years. (3) Complete clinical data. Exclusion criteria: (1) Secondary hypertension. (2) Malignancy or severe hepatic/renal dysfunction (serum creatinine ≥ 442 μmol/L). (3) Congenital or valvular heart disease. (4) Acute cardio-cerebrovascular events within the past 3 months. (5) Psychiatric or cognitive disorders hindering compliance. Patients were randomly divided into training (*n* = 295) and validation (*n* = 126) sets at a 7:3 ratio (12). The study was approved by the hospital ethics committee, with written informed consent obtained from all participants.

### Data collection

Demographics (age, sex, hypertension duration, smoking status, and diabetes history) were recorded. Biochemical and molecular markers were assayed: (1) Catecholamines (norepinephrine [NE], epinephrine [E], normetanephrine [NMN], metanephrine [MN]) via high-performance liquid chromatography. (2) SUA, serum creatinine, total cholesterol, LDL-C, and cystatin C via automated biochemical analyzer. Blood pressure parameters: 24h ambulatory blood pressure monitoring-derived mean SBP, SBP variability, and nocturnal mean DBP. Target organ structural indices: left ventricular posterior wall thickness (echocardiography); carotid intima-media thickness (ultrasound).

Definition of Organ Damage (≥1 of the following): (1) LV posterior wall thickness ≥11 mm; (2) Carotid IMT ≥ 1.0 mm; (3) Serum creatinine ≥ 115 μmol/L (men) or ≥ 107 μmol/L (women); (4) Cystatin C ≥ 1.25 mg/L (13).

### Statistical analysis

Data were analyzed using SPSS 26.0 and R 4.2.1. Continuous variables were expressed as mean ± standard deviation (x̄ ± SD) (independent t-test); categorical variables as n (*χ²* test). Candidate predictors (*P* < 0.05 in univariate analysis) were entered into multivariate logistic regression (stepwise regression method) to identify independent factors, and their odds ratios (OR) and 95% confidence intervals (CI) were calculated. A nomogram was constructed based on independent predictors. The receiver operating characteristic (ROC) curve was plotted, and the area under the curve (AUC) value was calculated. A model was considered to have good accuracy when the AUC value was between 0.7 and 0.9 and extremely high accuracy when >0.9. A calibration curve was plotted and evaluated using the Hosmer-Lemeshow goodness-of-fit test. The closer the calibration curve was to the 45-degree diagonal and the *P*-value of the H-L test > 0.05, the better the consistency between the predicted probability of the model and the actual incidence. Decision curve analysis (DCA) was used to evaluate the clinical application value of the nomogram by calculating the net benefit at different threshold probabilities.

## Results

### Baseline characteristics comparison between training and validation sets

No significant differences (*P* > 0.05) were observed in age, catecholamines (NE, E, NMN, MN), SUA, BP parameters, target organ indices, or comorbidities (diabetes, smoking history) between training and validation sets, confirming comparability (**Table 1**).

**Table 1:** Baseline characteristics of hypertensive patients in training and validation sets.

Indicator	Training set (*n* = 295)	Validation set (*n* = 126)	*t*/χ²	*P*
Age (years)	49.06 ± 9.67	50.51 ± 9.86	1.401	0.162
Norepinephrine (NE, ng/ml)	306.09 ± 82.17	308.95 ± 76.21	0.334	0.739
Epinephrine (E, ng/ml)	40.21 ± 14.37	42.12 ± 15.21	1.227	0.221
Normetanephrine (NMN, pg/ml)	181.72 ± 47.13	186.25 ± 45.21	0.914	0.361
Metanephrine (MN, pg/ml)	56.88 ± 19.39	58.62 ± 21.25	0.819	0.413
Serum Uric Acid (μmol/L)	388.14 ± 77.39	399.52 ± 67.52	1.434	0.152
24-hour Mean SBP (mmHg)	140.29 ± 13.89	143.22 ± 15.85	1.898	0.058
24-hour SBP CV	0.14 ± 0.05	0.15 ± 0.06	1.766	0.078
Nocturnal Mean DBP (mmHg)	80.54 ± 8.88	81.53 ± 8.95	1.045	0.297
LV Posterior Wall Thickness (mm)	9.95 ± 1.68	9.98 ± 1.95	0.159	0.873
Carotid IMT (mm)	0.99 ± 0.19	1.01 ± 0.21	0.958	0.339
Serum Creatinine (μmol/L)	81.73 ± 19.49	82.35 ± 18.61	0.302	0.763
Diabetes History (Yes/No)	105/190(35.59/64.41)	46/80(36.51/63.49)	0.032	0.858
Hypertension Duration (years)	10.43 ± 4.54	11.21 ± 5.12	1.553	0.121
Smoking History (Yes/No)	98/197(33.22/66.78)	40/86(31.75/68.25)	0.087	0.768
Total Cholesterol (mmol/L)	5.25 ± 1.12	5.42 ± 1.21	1.392	0.165
LDL-C (mmol/L)	3.56 ± 0.89	3.66 ± 0.95	1.035	0.302
Cystatin C (mg/L)	1.09 ± 0.31	1.12 ± 0.35	0.874	0.383

*t*: t-statistic of the independent t-test (for continuous variables), χ²: chi-square statistic of the chi-square test (for categorical variables), *P* < 0.05 indicates a statistically significant difference between the two sets.

Univariate analysis of factors associated with target organ damage in hypertensive patients in training set.

In training set, 130 (44.07%) hypertensive patients with TOD were observed, and 165 (55.93%) without TOD were observed. Univariate analysis showed statistically significant differences between the two groups in the following parameters: norepinephrine, normetanephrine, metanephrine, serum uric acid, serum creatinine, duration of hypertension, and cystatin C (all *P* < 0.05) (**Table 2**).

**Table 2:** Univariate analysis of factors associated with target organ damage in hypertensive patients in training set.

Indicator	TOD group (*n* = 130)	Non-TOD group (*n* = 165)	*t*/χ²	*P*
Age (years)	50.12 ± 9.81	48.23 ± 9.51	1.671	0.096
Norepinephrine (NE, ng/ml)	318.42 ± 92.51	296.33 ± 71.81	2.309	0.022
Epinephrine (E, ng/ml)	41.21 ± 16.72	39.42 ± 12.19	1.063	0.289
Normetanephrine (NMN, pg/ml)	189.62 ± 52.32	175.51 ± 41.72	2.577	0.011
Metanephrine (MN, pg/ml)	61.51 ± 21.42	53.23 ± 16.82	3.719	0.001
Serum Uric Acid (μmol/L)	402.51 ± 85.31	376.82 ± 68.72	2.865	0.005
24-hour Mean SBP (mmHg)	141.61 ± 15.22	139.33 ± 12.71	1.402	0.162
24-hour SBP CV	0.15 ± 0.04	0.14 ± 0.05	1.859	0.064
Nocturnal Mean DBP (mmHg)	81.42 ± 9.62	79.85 ± 8.21	1.511	0.132
LV Posterior Wall Thickness (mm)	10.12 ± 1.85	9.82 ± 1.51	1.533	0.126
Carotid IMT (mm)	1.01 ± 0.23	0.97 ± 0.17	1.717	0.087
Serum Creatinine (μmol/L)	88.61 ± 21.52	76.31 ± 15.81	5.657	0.001
Diabetes History (Yes/No)	50/80 (28.46/61.54)	55/110 (33.33/66.67)	0.834	0.361
Hypertension Duration (years)	11.21 ± 5.32	9.81 ± 3.73	2.653	0.008
Smoking History (Yes/No)	48/82 (36.92/63.08)	50/115 (30.31/69.69)	1.436	0.231
Total Cholesterol (mmol/L)	5.32 ± 1.21	5.21 ± 1.05	0.835	0.404
LDL-C (mmol/L)	3.62 ± 0.91	3.52 ± 0.88	0.955	0.341
Cystatin C (mg/L)	1.25 ± 0.32	0.98 ± 0.25	8.137	0.001

*t*: t-statistic of the independent t-test (for continuous variables), χ²: chi-square statistic of the chi-square test (for categorical variables), *P* < 0.05 indicates a statistically significant difference between the two groups.

Multivariate logistic regression analysis of organ damage in hypertensive patients Organ damage (Yes = 1, No = 0) was set as the dependent variable, and covariates with *P* < 0.05 in univariate analysis were incorporated into the multivariate logistic regression model. The results demonstrated that norepinephrine (*OR* = 1.003, 95% *CI*: 1.001–1.007), normetanephrine (*OR* = 1.009, 95% *CI*: 1.002–1.015), metanephrine (*OR* = 1.026, 95% *CI*: 1.010–1.042), serum uric acid (*OR* = 1.003, 95% *CI*: 1.003–1.007), serum creatinine (*OR* = 1.036, 95% *CI*: 1.020–1.053), duration of hypertension (OR = 1.088, 95% CI: 1.021–1.160), and cystatin C (*OR* = 12.367, 95% *CI*: 4.136–39.977) were independent risk factors for organ damage in hypertensive patients (all *P* < 0.05) (**Table 3**).

**Table 3:** Multivariate logistic regression analysis of organ damage in hypertensive patients.

Item	β	SE	Wald	P	OR	95% *CI*
Norepinephrine	0.003	0.002	3.640	0.046	1.003	1.001–1.007
Normetanephrine	0.009	0.003	7.149	0.007	1.009	1.002–1.015
Metanephrine	0.025	0.008	10.182	0.001	1.026	1.010–1.042
Serum uric acid	0.003	0.002	2.139	0.044	1.003	1.003–1.007
Serum creatinine	0.035	0.008	18.801	0.001	1.036	1.020–1.053
Duration of hypertension	0.084	0.033	6.673	0.010	1.088	1.021–1.160
Cystatin C	2.508	0.560	19.246	0.001	12.367	4.136–39.977

β: Regression coefficient; SE, Standard error of the regression coefficient; Wald, Wald statistic for testing the significance of regression coefficient; *P* < 0.05 indicates a statistically significant difference; OR, Odds ratio, representing the relative risk of organ damage when the independent variable changes; 95% CI, 95% confidence interval of odds ratio.

### Construction of the nomogram prediction model

**Figure 1:**
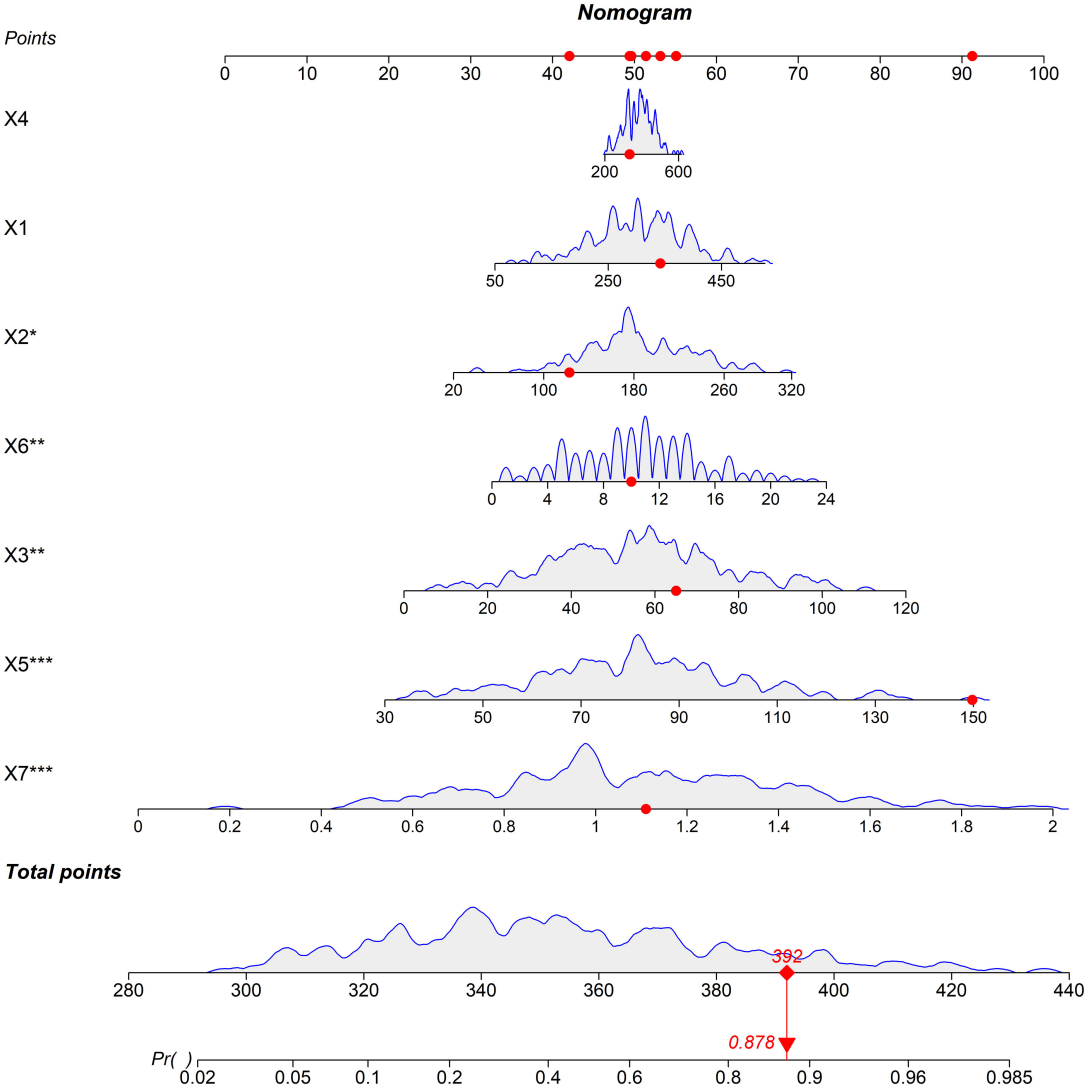
Nomogram prediction model for predicting the risk of organ damage in hypertensive patients (Note: X1: Norepinephrine, X2: Normetanephrine, X3: Metanephrine, X4: Serum uric acid, X5: Serum creatinine, X6: Duration of hypertension, X7: Cystatin C).

Based on the independent risk factors identified by multivariate logistic regression, a nomogram model was constructed. Each variable was assigned a corresponding score based on the standardized regression coefficients from the multivariate logistic regression analysis. The specific assignment method was as follows: first, the regression coefficients of each independent risk factor (norepinephrine, normetanephrine, metanephrine, serum uric acid, serum creatinine, duration of hypertension, and cystatin C) were standardized; then, a base score (0 points for the minimum value of each factor within the normal clinical range) was set, and the score for each factor level was calculated according to the standardized coefficients. These scores carry clinical significance: they quantify the contribution of each factor to hypertensive organ damage (higher scores mean stronger predictive effects), and the total score (sum of all factor scores) directly corresponds to the predicted risk of organ damage (a total score of 392 may correspond to 87.8% predicted risk). The total score was associated with the predicted risk of organ damage of hypertensive patients; a higher total score indicated a greater predicted risk of organ damage (**Figure 1**).

### Evaluation and validation of the nomogram model

**Figure 2:**
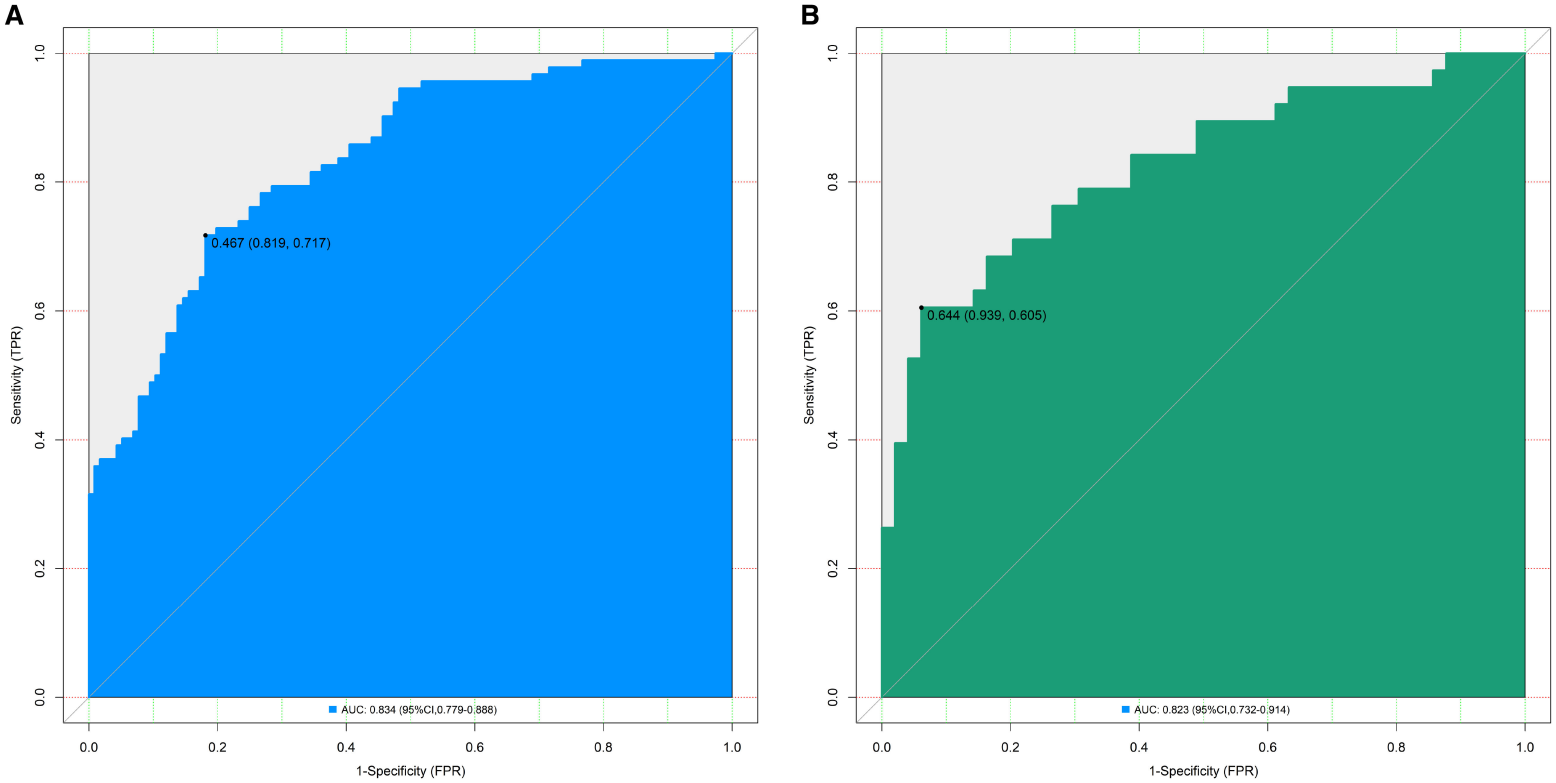
Receiver operating characteristic curves for predicting the risk of organ damage in hypertensive patients in the training set **(A)** and the validation set **(B)** (AUC: Area under the receiver operating characteristic curve (reflects model discriminative ability, AUC > 0.8 indicates excellent discriminative ability); 95% CI: 95% confidence interval; The values in parentheses represent (Specificity, Sensitivity), which respectively indicate the proportion of correctly identified non-organ damage patients and organ damage patients by the model).

**Figure 3:**
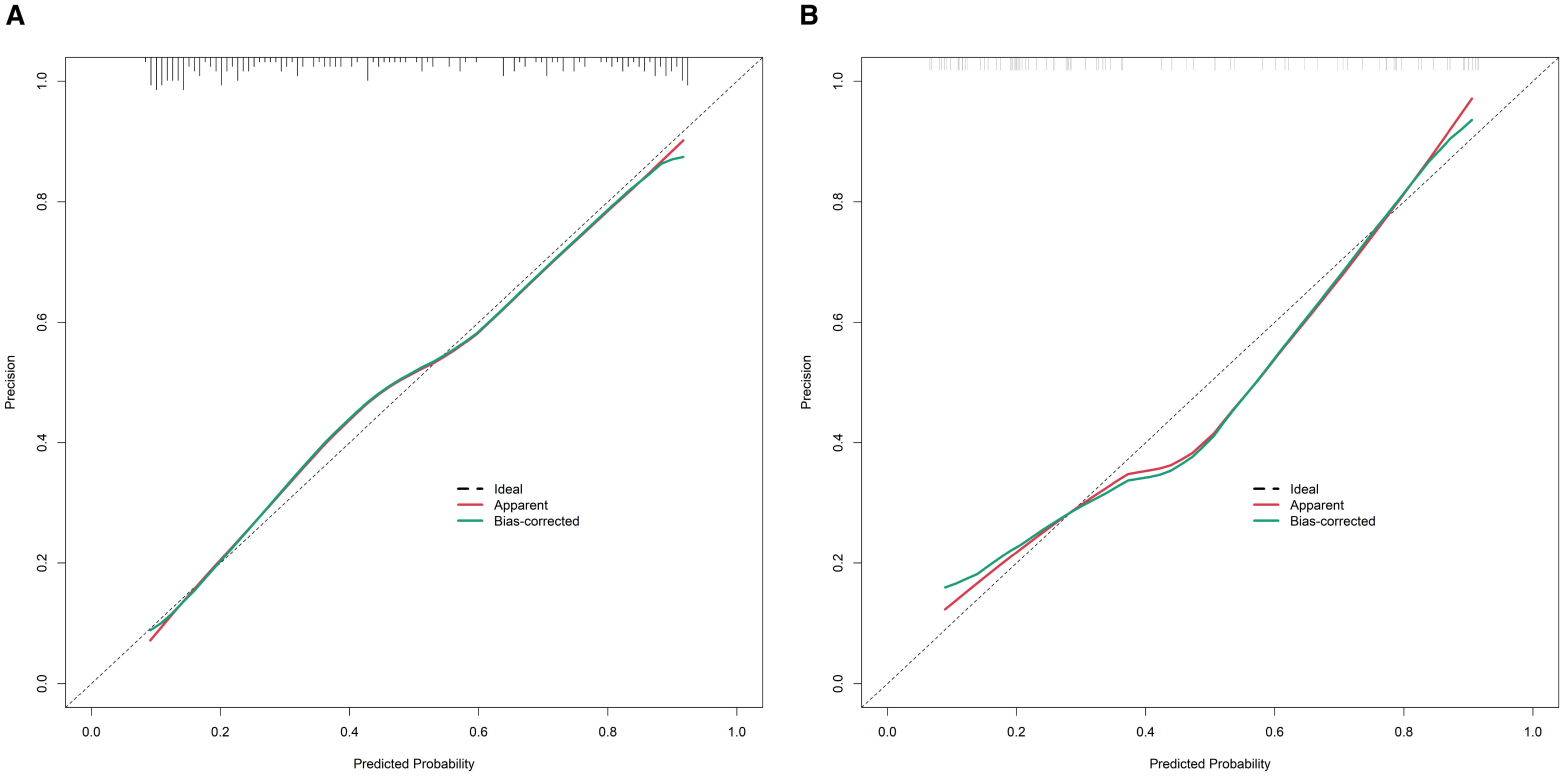
Calibration curves for predicting the risk of organ damage in hypertensive patients in the training set **(A)** and the validation set **(B)** (X-axis: the predicted probability of organ damage by the model, Y-axis: the actual observed probability of organ damage; The ‘Ideal’ line: perfect calibration (predicted probability = actual probability); The ‘Apparent’ line: the calibration of the original model; The ‘Bias-corrected’ line: the calibration after bias correction).

In the training set, the nomogram model exhibited a C-index of 0.834, and the Hosmer-Lemeshow test yielded *P* = 0.617, indicating good model fit. The ROC curve analysis revealed an AUC of 0.834 (95% *CI*: 0.779–0.888), with a sensitivity of 0.717 and specificity of 0.819. In the validation set, the C-index was 0.823, the Hosmer-Lemeshow test *P* = 0.472, and the AUC was 0.823 (95% *CI*: 0.732–0.914), with a sensitivity of 0.711 and specificity of 0.776. The ROC and calibration curves are displayed in **Figures 2** and **3**.

### Decision curve analysis

**Figure 4:**
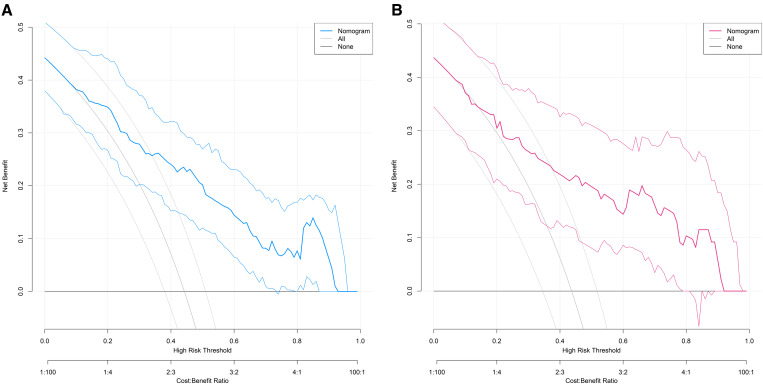
Decision curves for predicting the risk of organ damage in hypertensive patients in the training set **(A)** and the validation set **(B)** (X-axis: the threshold probability (minimum organ damage probability for clinical intervention), Y-axis: the net benefit (corrected difference between true positive and false positive proportions), ‘Nomogram’ curve: The net benefit of the proposed model, ‘None’ curve: The net benefit of the strategy of ‘predicting no organ damage for all patients’, ‘All’ curve: The net benefit of the strategy of ‘predicting organ damage for all patients’).

The decision curve demonstrated that the net benefit of the present nomogram model was significantly higher than the “predict all damage” or “predict no damage” strategies when the threshold probability ranged from 0.10 to 0.95, indicating its practical utility in clinical decision-making (**Figure 4**).

## Discussion

Hypertension, as a highly prevalent chronic disease worldwide, leads to target organ damage (e.g., cardiac hypertrophy, renal impairment, and carotid artery lesions), which serves as a core driver of cardiovascular events and mortality. According to statistics, the prevalence of hypertension among adults in China reaches 23.2%, and approximately 30%–50% of patients develop at least one organ injury within five years of diagnosis. Early identification of subclinical organ damage can reduce cardiovascular risk by 20%–30% (14, 15). However, conventional diagnostic methods exhibit significant limitations: ultrasonography is operator- and equipment-dependent, while biochemical markers such as serum creatinine only exhibit significant elevation when renal function loss exceeds 50%, indicating insufficient sensitivity and timeliness (16, 17). In this context, the present study aimed to integrate catecholamine-related markers (indicators of sympathetic nervous activity), serum uric acid (a marker of inflammation and metabolic dysfunction), and clinical parameters to establish a multimodal prediction model for improving the early detection of hypertensive organ damage. The results demonstrated that the nomogram model, incorporating norepinephrine, normetanephrine, metanephrine, serum uric acid, serum creatinine, hypertension duration, and cystatin C, achieved C-indices of 0.834 and 0.823 in the training and validation sets, respectively, with AUC values exceeding 0.8 and satisfactory sensitivity and specificity. Calibration curves and the Hosmer-Lemeshow test confirmed high consistency between predicted and observed values, while decision curve analysis indicated significant clinical net benefit, suggesting robust potential for clinical application.

The limited predictive efficacy of individual biomarkers in assessing hypertensive organ damage has been a key motivation for developing multimodal models. Previous studies reported that serum uric acid alone yielded an AUC of only 0.65–0.70 for predicting renal impairment, while catecholamines such as norepinephrine achieved an AUC of approximately 0.72–0.75 in predicting cardiac hypertrophy—neither of which meets clinical demands for early detection. By integrating multidimensional parameters, this study overcame the limitations of single-biomarker approaches, with its advantages manifesting in three aspects: first, the complementary nature of selected indicators. Catecholamines (norepinephrine, normetanephrine, and metanephrine) reflect sympathetic overactivation, promoting target organ damage through sustained vasoconstriction and enhanced oxidative stress. Serum uric acid contributes to renal and vascular injury via crystal deposition and inflammatory responses. Serum creatinine and cystatin C directly reflect renal function, with the latter increasing during mild renal insufficiency, compensating for the delayed elevation of creatinine. Hypertension duration captures the cumulative effect of prolonged blood pressure exposure on organ damage. These indicators collectively cover neuroregulatory, metabolic, renal functional, and chronic exposure mechanisms, forming a synergistic predictive framework.

Second, the robust performance of the model. The seven independent predictors identified via multivariable logistic regression demonstrated stable predictive value in both the training and validation sets. The high C-index (0.834 vs. 0.823) and AUC (0.834 vs. 0.823) (>0.8) indicated excellent discriminative ability to distinguish patients with and without organ damage. Balanced sensitivity (0.717, 0.711) and specificity (0.819, 0.776) suggested reduced risks of both false negatives and false positives, making the model suitable for clinical screening.

Third, clinical practicality. As a visual tool, the nomogram transformed complex regression equations into an intuitive scoring system, enabling clinicians to rapidly estimate the probability of organ damage based on seven parameters without intricate calculations—an advantage in primary care settings. Calibration curves confirmed minimal deviation between predicted and observed risks (Hosmer-Lemeshow test: P > 0.05), while decision curve analysis demonstrated significant net benefit at threshold probabilities of 0.10–0.95, indicating that model-guided interventions could optimize resource allocation while minimizing missed diagnoses.

Multivariable logistic regression confirmed norepinephrine, normetanephrine, metanephrine, serum uric acid, serum creatinine, hypertension duration, and cystatin C as independent predictors of hypertensive organ damage. The primary catecholamine released by sympathetic nerves, NE induces vasoconstriction via α-receptor activation, increasing peripheral resistance and cardiac afterload, thereby promoting left ventricular hypertrophy. Concurrently, NE-mediated oxidative stress accelerates carotid intima-media thickening (18). Each 1 ng/ml increase in NE elevated organ damage risk by 0.3% (*OR* = 1.003), aligning with prior evidence that sympathetic overactivation is a central mechanism of hypertensive organ injury (19). As stable metabolites of NE and epinephrine, these markers reflect long-term sympathetic activity (20). Each 1 pg/ml increase in NMN and MN raised organ damage risk by 0.9% (*OR* = 1.009) and 2.6% (*OR* = 1.026), respectively, with MN’s higher OR suggesting its greater predictive weight, possibly due to its association with renal hemodynamic dysfunction (21). Hyperuricemia contributes to renal inflammation, oxidative stress, and vascular smooth muscle proliferation, exacerbating carotid and cardiac damage (22). Each 1 μmol/L increase conferred a 0.3% higher risk (*OR* = 1.003), consistent with its established role as an independent risk factor for renal and cardiovascular events. Notably, catecholamines and uric acid may synergistically exacerbate organ damage via a vicious cycle (23, 24). Serum creatinine elevated creatinine indicates glomerular filtration decline. Each 1 μmol/L increase increased risk by 3.6% (*OR* = 1.036, 95% CI: 1.020–1.053, ***P*** = 0.001), underscoring renal impairment as both a manifestation and amplifier of organ damage. Among the risk factors, serum creatinine was assigned a score of >90. Although serum creatinine has insufficient sensitivity in predicting early renal function loss (only significantly elevated when renal function loss exceeds 50%), it was still included in the model and given a relatively high score for the following reasons: first, it is a classic, widely used, and standardized clinical indicator of renal function, ensuring data reliability; second, it has high specificity for moderate to severe renal function damage, and multivariate regression confirmed it as an independent risk factor for organ damage; third, it complements cystatin C (sensitive to early renal impairment) to cover the detection of both early and moderate to severe renal damage, improving the model’s comprehensive predictive ability. Superior to creatinine, cystatin C detects early renal dysfunction unaffected by muscle mass or diet (25). Its exceptionally high *OR* (12.367) per 1 mg/L increase highlights its sensitivity for early-stage injury (26). Each additional year of hypertension raised risk by 8.8% (OR = 1.088), reflecting cumulative vascular remodeling and fibrosis (27–29).

This study’s strengths include (1) the innovative integration of catecholamines, uric acid, and clinical parameters, covering neuro-metabolic-renal-chronic exposure mechanisms to achieve superior predictive performance (AUC > 0.8); and (2) high clinical translatability, with a user-friendly nomogram suitable for broad healthcare settings.

However, this study also has some limitations. Firstly, the model is only validated internally, and it is crucial to use independent queues for external validation in the future to confirm universality. And the exclusion of inflammatory/oxidative stress markers. Future multicenter prospective studies are warranted. This study is a cross-sectional study, and caution should be exercised when extrapolating conclusions.

In conclusion, this multimodal nomogram effectively predicts hypertensive organ damage by integrating seven mechanistically relevant indicators. Despite limitations, it offers a novel tool for early detection and intervention.

## Data Availability

The original contributions presented in the study are included in the article/supplementary material. Further inquiries can be directed to the corresponding author.
